# Selective Insulin Resistance in the Kidney

**DOI:** 10.1155/2016/5825170

**Published:** 2016-05-09

**Authors:** Shoko Horita, Motonobu Nakamura, Masashi Suzuki, Nobuhiko Satoh, Atsushi Suzuki, George Seki

**Affiliations:** ^1^Department of Internal Medicine, The University of Tokyo Hospital, 7-3-1 Hongo, Bunkyo, Tokyo 113-8655, Japan; ^2^Yaizu City Hospital, 1000 Dobara, Yaizu, Shizuoka 425-8505, Japan

## Abstract

Insulin resistance has been characterized as attenuation of insulin sensitivity at target organs and tissues, such as muscle and fat tissues and the liver. The insulin signaling cascade is divided into major pathways such as the PI3K/Akt pathway and the MAPK/MEK pathway. In insulin resistance, however, these pathways are not equally impaired. For example, in the liver, inhibition of gluconeogenesis by the insulin receptor substrate (IRS) 2 pathway is impaired, while lipogenesis by the IRS1 pathway is preserved, thus causing hyperglycemia and hyperlipidemia. It has been recently suggested that selective impairment of insulin signaling cascades in insulin resistance also occurs in the kidney. In the renal proximal tubule, insulin signaling via IRS1 is inhibited, while insulin signaling via IRS2 is preserved. Insulin signaling via IRS2 continues to stimulate sodium reabsorption in the proximal tubule and causes sodium retention, edema, and hypertension. IRS1 signaling deficiency in the proximal tubule may impair IRS1-mediated inhibition of gluconeogenesis, which could induce hyperglycemia by preserving glucose production. In the glomerulus, the impairment of IRS1 signaling deteriorates the structure and function of podocyte and endothelial cells, possibly causing diabetic nephropathy. This paper mainly describes selective insulin resistance in the kidney, focusing on the proximal tubule.

## 1. Introduction

Insulin is one of the essential hormones in humans [1]. It is secreted from pancreatic *β* cells and regulates glucose homeostasis in various organs and tissues, such as the liver, muscle and fat tissues, and the kidney. However, the roles of insulin in these organs and tissues are pleiotropic and variable. In the liver, insulin inhibits gluconeogenesis, promotes glycogen synthesis, and activates* de novo* lipogenesis. In the muscle and adipocytes, insulin stimulates glucose uptake [2].

**Figure 1:**
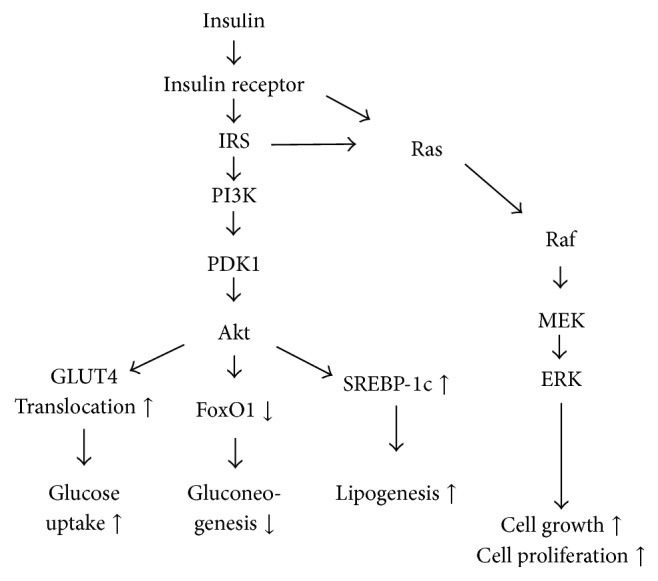
Typical insulin signaling cascade. Insulin binds to insulin receptor in the cell surface [7, 13–15]. The signal goes via IRS, PI3K, and PDK1 to Akt. Akt is a key regulator of this cascade and triggers various signals of physiological responses such as stimulation of glucose uptake, inhibition of gluconeogenesis, and stimulation of lipogenesis. Insulin also stimulates cell growth and proliferation via MEK and ERK cascade. IRS: insulin receptor substrate, PI3K: isoform of phosphatidylinositol 3′-kinase, PDK1: 3′-phosphoinositide-dependent protein kinase-1, GLUT4: glucose transporter type 4, FoxO1: Forkhead box protein O1, and SREBP-1c: sterol regulatory element-binding protein 1c.

Insulin exerts its activity via signal transduction pathways that start from the binding of insulin to the insulin receptor (IR) [3]. Via insulin receptor substrate (IRS), the signal is transmitted to phosphoinositide 3-kinase (PI3K) and phosphoinositide-dependent kinase-1 (PDK1) and leads to the phosphorylation of Akt. The phosphorylated Akt mediates vital signals such as stimulating protein synthesis, cell survival, transcription, and glycogen synthesis [4]. There are four subtypes of IRS (IRS1 to IRS4), among which IRS1 and IRS2 are the primary mediators involved in insulin signaling [5]. There are other signal transduction pathways initiated by insulin, such as mitogen-activated protein kinase (MAPK)/extracellular signal-regulated kinase (ERK) kinase (MEK) and ERK, which, partly under IRS-mediated signal and partly under Shc that directly mediates signals from the IR, serve to primarily regulate cell growth and proliferation [6, 7].  Figure 1 shows the simplified scheme of the insulin signal transduction network.

However, in insulin resistance, the target organs and/or tissues do not fully respond to insulin. Insulin resistance is characterized by attenuation of the insulin effect. The problem is that the phenotype of insulin resistance is complex; for example, liver insulin receptor knockout mice (LIRKO) show hyperglycemia and hypolipidemia, due to total liver insulin signal deficiency [8]. In contrast, in humans and in animal models of metabolic syndrome-induced insulin resistance, there is hyperglycemia and hyperlipidemia caused by preserved lipogenesis [9]. Additionally, the mechanisms of insulin resistance is different among organs and tissues. For example, in adipose tissue, both IRS1-dependent and IRS2-dependent signals are impaired in insulin resistance. However, in the renal proximal tubule (PT), insulin signaling via IRS1 is impaired but insulin signaling via IRS2 is preserved [10–12].

## 2. Selective Insulin Resistance in the Liver and Vascular Endothelium

As shown in Figure 1, the insulin signaling cascade has various effects on glucose and lipid metabolism. The insulin signaling cascade mediates signals for gluconeogenesis inhibition by inhibiting the Forkhead box protein O1 (FoxO1) activity [22] and for activation of glucose uptake by inducing glucose transporter 4 (GLUT4) translocation to the plasma membrane [23]. The insulin signaling cascade also induces lipogenesis by activating sterol regulatory element-binding protein- (SREBP-) 1c [13, 24]. In insulin resistance, these effects are impaired to different extents.

In the liver, IRS1 mediates lipogenesis while IRS2 mediates glycogen synthesis. In insulin resistance, the signaling cascade via IRS2 seems to be impaired while the signaling cascade via IRS1 seems relatively intact [25, 26]. Furthermore, in hepatocytes with insulin resistance, SREBP-1c expression is increased, while the expressions of IRS2 and insulin-induced Akt phosphorylation are significantly decreased [26]. The IRS1-dependent pathway is essential for SREBP-1c expression triggered by insulin [25], while the PI3K signaling that is dependent on IRS2 has been shown to be essential for enhancement of glucose uptake by inhibiting glycogen synthase kinase (GSK) 3*β* [27]. These results suggest that both gluconeogenesis and lipogenesis are stimulated under hepatic insulin resistance, inducing hyperglycemia and hyperlipidemia. The difference between the role of IRS1 and IRS2 in insulin signaling cascade could account for the existence of selective insulin resistance in liver. In fact, the liver is the first organ in which selective insulin resistance was noticed [28–30].

In the liver, the evidence of specific zonation for metabolic pathways [31–34] has accumulated. Two zones, an afferent periportal area and an efferent perivenous region, are known so far. The periportal, upstream area is supplied with blood rich in oxygen and hormones such as insulin and is involved in oxidative energy metabolism and gluconeogenesis. On the other hand, the perivenous downstream area is supplied with blood which is poor in oxygen but rich in CO_2_ and metabolic products and is engaged in glucose uptake and lipogenesis [34–36]. Recent studies suggest that selective insulin resistance might be related to hepatic zonation; the periportal area becomes insulin resistant, while the perivenous area remains insulin sensitive and thus accompanied with enhanced gluconeogenesis and retained lipogenesis [37].

In vascular endothelial cells, IR/IRS1/PI3K/Akt pathways are thought to be attenuated in insulin resistance, while the ERK/MAPK pathways are not affected in obese Zucker rats [38], suggesting the existence of selective insulin resistance. Insulin increases endothelial nitric oxide (NO) production and endothelial NO synthase (eNOS) gene expression via PI3K and Akt, which is impaired in insulin resistance [39, 40]. The gene expression of eNOS is altered by IRS1 [41]. Mice lacking IRS1 present impaired endothelium-dependent vascular relaxation, suggesting that IRS1 plays a significant role in insulin signal transduction at the vascular endothelium [42]. IRS2^−/−^ mice exhibit more intensive neointima formation compared to wild type and IRS1^−/−^ mice [43]. This attenuation of vascular insulin signaling appears to mainly involve the IRS1-dependent pathway but IRS2-dependent pathway may also be partly involved. On the other hand, preserved ERK/MAPK pathway triggers the expression of endothelin-1, a strong vasoconstrictor that leads to blood pressure elevation [44]. Additionally, the lack of insulin receptor gene and resulting attenuation of insulin signaling were shown to increase the expression of vascular cell adhesion molecule 1 (VCAM-1) [38, 44, 45], a strong inducer of atherosclerosis, showing that loss of insulin signaling could accelerate atherosclerosis. Taken together, selective insulin resistance also exists in the vascular endothelium; that is, the attenuation of IRS/PI3K pathway could impair NO-derived vasodilatation and promote atherosclerosis, while the preserved ERK/MAPK pathway could induce vasoconstriction mediated by endothelin-1.

## 3. The Roles of IRS1 and IRS2 in Muscle and Adipose Tissue in Insulin Resistance

In muscle, insulin signaling via IRS and PI3K is attenuated, whereas the stimulation of the MAPK pathway by insulin is thought to be intact in hyperinsulinemia and type 2 diabetes mellitus (T2DM) [46–48]. Huang and colleagues showed that gene silencing of IRS1, but not IRS2, in L6 myotube cells reduced GLUT4 translocation and glucose uptake, suggesting that IRS1 is mainly involved in insulin-stimulated glucose uptake [49]. Other studies also suggest that muscle IRS1, but not IRS2, is reduced in insulin resistance [50]. In muscle, insulin resistance seems to attenuate predominantly IRS1-dependent GLUT4 translocation and glucose uptake, though some reports suggest that IRS2-dependent insulin signaling cascade could also be involved in glucose metabolism in myocytes [51, 52].

Adipose tissue is the major site responsible for systemic insulin resistance. Ueki and colleagues showed that suppressor of cytokine signaling 1 (SOCS-1) and SOCS-3 causes impaired insulin signaling through the phosphorylation of IRS1 and IRS2 by separate mechanisms [53]. In human adipocytes, exposure to glucose and high dose insulin for several hours reduced IRS1 expression [54] while, in rat adipocytes, high fat diet reduced both IRS1 and IRS2 content [55, 56]. The reason why IRS1 and IRS2 are not suppressed identically between human and rat adipocytes is not understood.

Insulin induces translocation of GLUT4, the main glucose transporter isoform expressed in adipocytes, from the cytosol to the plasma membrane [57, 58]. Overexpression of human IRS1 in rat adipocytes elevates surface GLUT4 level even without insulin [59]. In insulin resistance, the adipocyte itself is enlarged and the expression of IRS1 and GLUT4 is decreased. Inflammation factors such as TNF*α* and IL-1*β* are thought to induce reduction of IRS1 and GLUT4 expression [60, 61].

## 4. Chronic Kidney Disease (CKD) and Insulin Resistance

Substantial evidence indicates that insulin resistance is accompanied by chronic kidney disease (CKD) [62]. Insulin resistance is frequently seen in patients with advanced or end-stage renal disease and also in patients with mild renal dysfunction [63, 64]. The mechanisms involved in the occurrence and development of insulin resistance in CKD has been clarified to some extent [62]. Inflammation is a notorious contributor to the emergence of insulin resistance. Mediators of chronic inflammation, such as TNF-*α*, IL-6, and interferon-*γ* show increased levels in CKD patients [65–67]. The binding of insulin to IR is well preserved, but the signal transduction cascade after insulin binding to IR seems to be impaired [68, 69]. The main inhibitory step is the degradation of IRS1 by the ubiquitin complex, which reduces phosphorylation of Akt that is downstream of IRS1, resulting in abnormal glucose homeostasis and lipid metabolism [70, 71].

## 5. Recent Findings regarding Insulin Signaling in the Renal Proximal Tubule

In renal tubules, insulin stimulates sodium reabsorption in many tubular segments [12, 72, 73]: the PT [74–77], thick ascending loop of Henle (TAL) [78, 79], distal convoluted tubule (DCT) [80], and cortical collecting duct (CCD) [81]. As for the PT, signal transduction cascade initiated by insulin triggers Akt phosphorylation, mainly via IRS2/PI3K [11, 82]. In the DCT, the insulin signal pathway includes Akt and with-no-lysine kinase (WNK) [83].

We have recently showed [11] that in PTs of the Otsuka Long Evans Tokushima Fatty (OLETF) rat, an animal model of insulin resistance, the stimulatory effect of insulin on sodium bicarbonate cotransporter (NBCe1) activity via the PI3K/Akt pathway was also totally intact. This strongly suggests that, even in insulin resistance, the enhancement of sodium reabsorption via NBCe1 by insulin is preserved. We also showed that, in the kidney cortex of insulin resistant OLETF rats, the expression of IRS1 was decreased to some extent but the expression of IRS2 was totally retained. Why the expression of IRS2 in the kidney cortex is preserved in insulin resistance is not completely understood. However, after feeding and insulin administration, the protein expression of steroid regulatory element-binding protein 1 (SREBP1) was decreased in the liver but preserved in the kidney cortex, whereas the protein expression of Forkhead box protein O1 (FoxO1) was elevated in the liver but unchanged in the kidney cortex. This indicates that the liver and kidney cortex have different regulatory mechanisms for IRS2 expression [11]. Others have also showed that the kidney expression of IRS2 was preserved even in diabetic rats [10]. These facts support our findings of different IRS2 expression regulation between kidney and liver. We also confirmed that this stimulatory effect of insulin on NBCe1 in the PT is preserved in humans with insulin resistance as well. These results suggest a possibility that hyperinsulinemia accompanied with insulin resistance is an important factor for the onset and progression of hypertension in metabolic syndrome and is mediated through the IRS2/PI3K/Akt signaling pathway.

We have also subsequently reported that, in OLETF rats with overt diabetic nephropathy accompanied with massive proteinuria, the stimulatory effect of insulin on renal proximal sodium reabsorption was preserved [16]. The expression of IRS2 and the insulin-induced phosphorylation of Akt in the kidney cortex were also preserved in these rats. Moreover, in the PT of human subjects with type 2 diabetic nephropathy, insulin significantly stimulated NBCe1 activity. These results indicate that the stimulatory effect of insulin on PT sodium reabsorption is preserved even in overt diabetic nephropathy with massive proteinuria. This could at least partially explain why intensive glycemic control for patients with T2DM and CKD is often complicated with massive weight gain, prolonged hyperinsulinemia, and hypoglycemia due to decreased renal function and poor prognosis due to increased cardiovascular risk [84].

In contrast, OLETF rats have a significantly decreased expression of IRS1 in the kidney cortex. This may be relevant to the fact that gluconeogenesis, which is restricted to the PT in the kidney, is enhanced in DM, and this is supported by control experimental animals having suppressed PT gluconeogenesis due to an intact IRS1 pathway [85–88]. In insulin resistance, diabetes, and overt diabetic nephropathy, IRS2-dependent stimulation of sodium transport by insulin in the PT is preserved and this may induce sodium retention, whereas IRS1-dependent suppression of gluconeogenesis is attenuated and might induce hyperglycemia. These findings suggest that insulin signaling is selectively impaired in the PT, under conditions of systemic insulin resistance, diabetes, and even overt diabetic nephropathy.

## 6. Recent Findings regarding Insulin Signaling in Glomeruli

The glomerulus is composed of three cell types: podocytes, endothelial glomerular cells, and mesangial cells. All of these cells have been shown to respond to insulin stimulation. In the glomerular endothelial cells, insulin can increase nitric oxide (NO) production by stimulating eNOS activity [39]. This effect seems to be impaired in animal models of insulin resistance and diabetes [89, 90]. In primary cultures, podocytes have the highest IR and IRS1 expression levels compared with endothelial cells and mesangial cells [10]. Insulin was suggested to play a role in the regulation of podocyte contractility, which may contribute to glomerular permeability [91, 92]. Podocyte-specific IR knockout mice develop albuminuria, the effacement of podocyte foot process, and podocyte apoptosis. These mice also have increased glomerular matrix level, glomerulosclerosis, and glomerular basement membrane (GBM) thickening, which recapitulates some features of diabetic nephropathy. This suggests that podocyte-specific insulin signaling is crucial for glomerular function [93]. Insulin is also reported to modulate glomerular permeability by controlling podocyte contractility [17]. Insulin exerts its effect on mesangial cells; insulin has been shown to inhibit mesangial cell apoptosis, by activating the PI3K pathway and enhancing mesangial cell proliferation [94–96].

Similarly in glomeruli, many reports suggest that insulin signaling is altered in insulin resistance and diabetes. Using animal models of insulin resistance and T2DM, Mima and colleagues showed an attenuation of glomerular IRS1 expression, IRS1 phosphorylation, and glomerular endothelial signaling. In contrast, IRS2 expression was preserved in these glomerular endothelial cells [10]. The glomerular insulin signaling cascade via Akt2 is thought to be crucial for the maintenance of glomerular function and structure [93, 97]. The impairment of IRS2 signaling in the podocyte in the onset of diabetic nephropathy has been very recently suggested; Santamaria and colleagues demonstrated that phosphatase and tensin homolog (PTEN) and IRS2 were essential for insulin signaling in podocytes [18].

These findings could help to elucidate the mechanisms of the emergence and the progression of diabetic nephropathy. The existence of insulin resistance in type 1 diabetes (T1D) is a remarkable risk factor for the progression to overt nephropathy [98–100]. Additionally, in various animal models of renal injury, thiazolidinediones (TZDs), used clinically for improving insulin sensitivity, are reported to have renoprotective effects on the glomeruli [101–103], suggesting that glomerular insulin resistance could be an important factor for the impairment of renal function.

**Figure 2:**
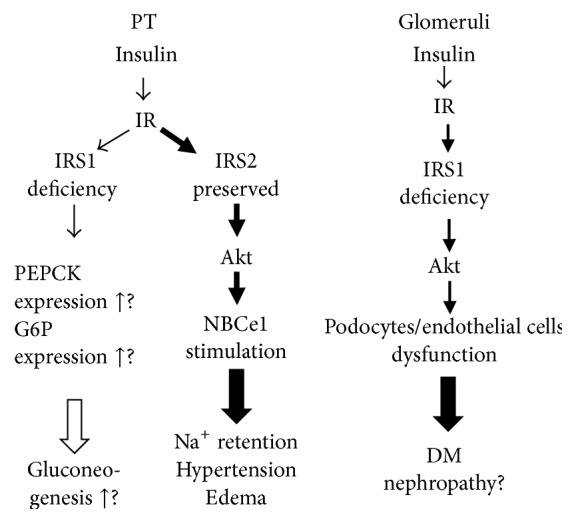
Insulin signaling in insulin resistance in the kidney [10, 11, 16–21]. In the PT, the expression of IRS1 is suppressed while that of IRS2 is preserved. IRS1 signaling deficiency may cause the upregulation of G6P and PEPCK. In this condition, the inhibition of gluconeogenesis does not work sufficiently, causing enhanced gluconeogenesis and hyperglycemia. On the other hand, the expression of IRS2 is preserved, causing stimulation of sodium reabsorption via NBCe1, leading to sodium retention, hypertension, and edema. In the glomeruli, IRS1 signaling is also impaired similar to the PT. This causes podocyte dysfunction, possibly leading to DM nephropathy. Both the PT and glomeruli have selective insulin resistance, with differences between signaling via IRS1 and signaling via IRS2. IR: insulin receptor, IRS: insulin receptor substrate, FoxO1: Forkhead box protein O1, NBCe1: sodium bicarbonate cotransporter type 1, G6P: glucose-6-phosphatase, and PEPCK: phosphoenolpyruvate carboxykinase.

Taken together, the attenuation of glomerular insulin signaling cascade accompanied with insulin resistance and DM could be related to the emergence and progression of diabetic nephropathy. Conversely, selective insulin resistance in the PT could result in the preservation of the stimulatory effect of insulin on sodium transport as well as the attenuation of inhibitory effect of insulin on gluconeogenesis. This may explain the pathogenetic mechanisms of hypertension accompanied with hyperinsulinemia, edema, and fluid retention as a complication of metabolic syndrome and intensive insulin treatment. Unsuppressed renal gluconeogenesis could also at least partially contribute to hyperglycemia in DM. Thus, the selective insulin resistance in the kidney seems to be a common mechanism linking all negative effects on the emergence and progression of diabetic nephropathy and other complications, in both glomeruli and renal tubules, making the prevention and therapy of diabetic nephropathy even more challenging. In the glomeruli, the decrease of IRS1-dependent signaling impairs the functions of glomerular cells, endothelial cells, podocytes, and mesangial cells. In the PT, IRS2-dependent stimulation of sodium reabsorption could cause hypertension and edema, while impaired IRS1-dependent signaling could induce unsuppressed gluconeogenesis, possibly contributing to hyperglycemia. TZDs are suggested to have protective effects on the glomeruli by ameliorating insulin sensitivity. However, in the PT, TZDs enhance sodium retention [104] and can abolish the beneficial effect of TZDs on glomeruli.  Figure 2 summarizes selective insulin resistance in the kidney.

## 7. Conclusion

This paper provides an overview of the recent findings regarding selective insulin resistance in the kidney. This condition of “selective insulin resistance” has been previously recognized, but it is only recently that the existence of renal “selective insulin resistance” and some of its detailed mechanism have started to be elucidated. The characteristics of insulin resistance differ among organs and tissues; in liver IRS2-mediated pathway is impaired, causing hyperglycemia while IRS1-mediated pathway is preserved to some extent, inducing hyperlipidemia. In the glomerulus, deficiency of IRS1-mediated pathway causes glomerular dysfunction and possibly contributes to diabetic nephropathy. In the PT, IRS2-mediated pathway is preserved and the stimulation of sodium reabsorption by insulin causes sodium retention and possibly subsequent hypertension, whereas the potential impairment of the IRS1-mediated pathway could lead to unsuppressed gluconeogenesis. These facts support the existence of selective insulin resistance in the kidney. TZDs, drugs for improvement of insulin sensitivity, enhance sodium retention in the renal tubules. “Selective insulin resistance” in diabetic nephropathy could explain the challenges of treatment for hypertension and congestive heart failure accompanied with diabetes. Future investigation targeted at the improvement of selective insulin resistance will be of significance for the treatment of diabetes and its complications.
